# Characterization of a short isoform of the kidney protein podocin in human kidney

**DOI:** 10.1186/1471-2369-14-102

**Published:** 2013-05-06

**Authors:** Linus A Völker, Eva-Maria Schurek, Markus M Rinschen, Judit Tax, Barbara A Schutte, Tobias Lamkemeyer, Denise Ungrue, Bernhard Schermer, Thomas Benzing, Martin Höhne

**Affiliations:** 1 Department 2 of Internal Medicine and Center for Molecular Medicine Cologne, University of Cologne, Kerpener Str. 62, 50937, Cologne, Germany; 2 Cologne Excellence Cluster on Cellular Stress Responses in Aging-Associated Diseases, University of Cologne, Cologne, Germany; 3 Systems Biology of Ageing Cologne (Sybacol), University of Cologne, Cologne, Germany

**Keywords:** Podocin, Isoform, Kidney glomerulus

## Abstract

**Background:**

Steroid resistant nephrotic syndrome is a severe hereditary disease often caused by mutations in the *NPHS2* gene. This gene encodes the lipid binding protein podocin which localizes to the slit diaphragm of podocytes and is essential for the maintenance of an intact glomerular filtration barrier. Podocin is a hairpin-like membrane-associated protein that multimerizes to recruit lipids of the plasma membrane. Recent evidence suggested that podocin may exist in a canonical, well-studied large isoform and an ill-defined short isoform. Conclusive proof of the presence of this new podocin protein in the human system is still lacking.

**Methods:**

We used database analyses to identify organisms for which an alternative splice variant has been annotated. Mass spectrometry was employed to prove the presence of the shorter isoform of podocin in human kidney lysates. Immunofluorescence, sucrose density gradient fractionation and PNGase-F assays were used to characterize this short isoform of human podocin.

**Results:**

Mass spectrometry revealed the existence of the short isoform of human podocin on protein level. We cloned the coding sequence from a human kidney cDNA library and showed that the expressed short variant was retained in the endoplasmic reticulum while still associating with detergent-resistant membrane fractions in sucrose gradient density centrifugation. The protein is partially N-glycosylated which implies the presence of a transmembranous form of the short isoform.

**Conclusions:**

A second isoform of human podocin is expressed in the kidney. This isoform lacks part of the PHB domain. It can be detected on protein level. Distinct subcellular localization suggests a physiological role for this isoform which may be different from the well-studied canonical variant. Possibly, the short isoform influences lipid and protein composition of the slit diaphragm complex by sequestration of lipid and protein interactors into the endoplasmic reticulum.

## Background

**Figure 1:**
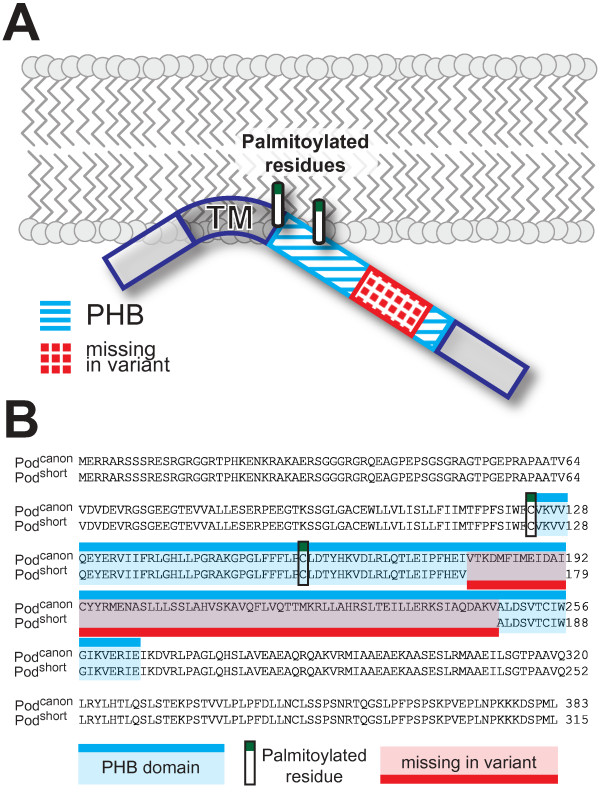
**Schematic overview and alignment of the podocin short isoform. A**) Schematic drawing of podocin with the difference between the two isoforms indicated. A major part of the PHB domain is missing in the short isoform. **B**) Sequence alignment of both human podocin isoforms, Pod^canon^ and Pod^short^. [UniProt:Q9NP85, UniProt:Q9NP85-2]. The PHB domain and the palmitoylated residues are indicated.

Podocin, the protein encoded by the *NPHS2* gene, is mutated in hereditary steroid-resistant nephrotic syndrome (SRNS) and essential for an intact kidney filtration barrier [1-3]. Podocin is expressed at the glomerular slit diaphragm in kidney podocytes, the visceral epithelial cells of the kidney filtration barrier. Knock-out mice have been shown to lack slit diaphragm and die within the first weeks of life due to kidney failure [3]. Human podocin consists of 383 amino acids and is predicted to form a hairpin-like structure within the inner leaflet of the plasma membrane lipid bilayer. A central PHB-domain and two palmitoylated residues enable podocin to recruit cholesterol [4]. Podocin’s function is currently understood as a scaffold providing the necessary protein-lipid microenvironment needed for proper function and signaling of the slit diaphragm protein complex [4,5]. The amino acid sequence is strongly conserved with approximately 86% identity between mouse and human homologues. Database searches and recent publications have suggested the presence of a shorter 315 amino acid isoform of human podocin that lacks one exon encoding the central part of the PHB-domain (Figure 1) [6,7]. However, all evidence so far has been based on antibody-mediated detection and RT-PCR, making it uncertain whether the protein is actually expressed. This is particularly interesting with regard to the importance of a functional PHB domain. Here we show that the short isoform is expressed in human kidneys and localizes to the endoplasmic reticulum raising the intriguing hypothesis that podocin may serve distinct functions in different parts of the podocyte.

## Methods

### Reagents and plasmids

The short isoform of human podocin was cloned with a nested PCR approach from a human kidney cDNA library. The first primer set is binding in the 5′ and 3′-UTR, respectively, and the second primer set is amplifying the ORF and adds cloning sites to the PCR construct (underlined in primer sequence). Primer sequences: hPod-19bp_fp: cgcccggcagctctgagga; hPod + 371bp_rp: ggctgtgggagctgtggcaa; hPod_mlu_fp: cgcgggacgcgtATGGAGAGGAGGGCGCGGAGC; hPod_not_rp: cgcggggcggccgccCTATAACATGGGAGAGTCTTTCTTTTTAGG. The ORF was cloned into a modified pcDNA6 expression vector which adds a V5-tag or a FLAG-tag, respectively, to the N-terminus of the expressed protein. To eliminate the putative N-glycosylation site at aa position 287 of the short isoform, a modified QuikChange site directed mutagenesis approach [8] was used with the following primer pair: hPod_NtoS_fp: ctgaattgcctgtcttctccgagctccagaactcagggaagcctc and hPod_NtoS_rp: gaggcttccctgagttctggagctcggagaagacaggcaattcag. All constructs were verified by sequencing.

The following IP buffer was used for cell lysis: 1% Triton X-100; 20 mM Tris pH 7.5; 25 mM NaCl; 50 mM NaF; 15 mM Na_4_P_2_O_7_; 1 mM EDTA; 0.25 mM PMSF; 5 mM Na_3_VO_4_.

Antibodies were obtained from Sigma (anti podocin #P0372), Serotec (anti V5 mAB #MCA1360), Millipore (anti V5 pAb #AB3792), and Santa Cruz (anti Flotillin-2 #sc-28320; anti-CD71/Transferrin receptor #sc-65882).

### Cell culture and transfection

HEK 293 T and HeLa cells were cultured in DMEM supplemented with 10% fetal bovine serum under standard conditions (5% CO_2_, 37°C). For transfection experiments, cells were grown to 60–80% confluency and transfected with plasmid DNA using the calcium phosphate method for HEK 293 T cells, or GeneJuice (Novagen) for HeLa cells according to the manufacturer’s instructions.

### Coimmunoprecipitation

Cells were incubated for 24 h after transfection, washed with PBS, lysed in ice-cold IP-buffer (see above) on ice for 15 Min and centrifuged (18.000 rpm, 4°C, 15 Min). Supernatants containing equal amounts of total proteins were incubated for 1 h at 4°C with M2 anti-FLAG agarose beads (Sigma). The beads were washed three times with IP-buffer and bound proteins were resolved by 10% SDS-PAGE.

### MS sample preparation

Human glomerular samples were prepared from healthy kidney tissue of tumor nephrectomies by a sieving technique described elsewhere [9]. The study protocol was approved by the independent ethics committee of Cologne University and all patients provided written informed consent.

Human glomerular samples were denatured by boiling in Lämmli buffer for 5 minutes, and unsoluble components pelleted by centrifugation. Soluble protein content was separated by standard SDS-Page gel electrophoresis (4-20% Gradient gel) and stained with colloidal Coomassie brilliant blue dye. Gel segments at the molecular weight expected for the podocin protein (canonical isoform: 42.2 kDa; short isoform: 34.4 kDa) were excised from the gel and subjected to mass spectrometry.

### Peptide isolation and mass spectrometry

#### Tryptic in-gel digest

Following electrophoresis the gel was washed thoroughly in water. The area of interest was cut out and minced using a scalpel. After destaining with 50% 10 mM NH_4_HCO_3_/50% ACN at 55°C and dehydration in 100% ACN gel pieces were equilibrated with 10 mM NH_4_HCO_3_ containing porcine trypsin (12.5 ng/μl; Promega) on ice for 4 hours. Excess trypsin solution was removed and tryptic hydrolysis was performed for 4 hours at 37°C in 10 mM NH_4_HCO_3_. The supernatant was collected and further extraction steps were performed. After acidification with 5% TFA, gel pieces were extracted twice with 1% TFA and then with 60% ACN/40%H_2_O/0.1% TFA followed by a subsequent two-step treatment using 100% ACN. The supernatant and the extractions were combined and concentrated using a SpeedVac concentrator (Christ). Prior to nano-LC-MS/MS analysis the peptides were desalted using STAGE Tip C18 spin columns (Proxeon/Thermo Scientific) as described elsewhere [10]. Eluted peptides were concentrated *in vacuo* and then re-suspended in 0.5% acetic acid in water to a final volume of 10 μl.

#### Nano-LC ESI-MS/MS mass spectrometry

Experiments were performed on a LTQ Orbitrap Discovery mass spectrometer (Thermo Scientific) that was coupled to an EASY-nLC nano-LC system (Proxeon/Thermo Scientific). Intact peptides were detected in the Orbitrap at 30,000 resolution in the mass-to-charge (m/z) range 350–2000. Internal calibration was performed using the ion signal of (Si(CH_3_)_2_O)_6_H at m/z 445.12003 as a lock mass. For LC-MS/MS analysis, up to five CID spectra were acquired following each full scan. Aliquots of the samples were separated on a 15 cm, 75 μm reversed phase column (Proxeon/Thermo Scientific). The gradient used for liquid chromatography is described elsewhere in more detail [11].

#### Peptide and protein identification

The search algorithm Sequest as implemented in the Proteome Discoverer software (Thermo Scientific) was used for protein identification. To identify the proteins contained in the excised gel area, MS/MS data were searched using the canonical sequence database of the *Homo sapiens* reference proteome provided by the UniProt Consortium using the target-decoy strategy. The sequence of the predicted short isoform of podocin was added to the database. The maximum of two modification was allowed per peptide. Oxidation of methionine residues was used as a variable modification and carbamidomethylation of cysteine residues as a fixed modification. For Orbitrap data, 10 ppm mass tolerance was allowed for intact peptide masses and 0.8 Da for CID fragment ions detected in the linear ion trap. Peptides were subsequently filtered to match a FDR < 0.01. Protein identifications were based on at least 2 peptides. Ion chromatograms were extracted using the NHLBI in-house software QUOIL which extracts ion chromatograms for identified peptides [12]. Isotope patterns were visualized using the MaxQuant Viewer software [13].

### Lipid raft preparation

The preparation of detergent resistant membrane domains (DRMs) was performed as described [14]. Briefly, HEK 293 T cells were homogenized in 1 ml lysis buffer (150 mM NaCl; 20 mM Tris/HCl pH 7.4; 0.1 mM EDTA; 1% Triton X-100; 5 mM Na_3_VO_4_; 0.25 mM PMSF) by 20 strokes in a Dounce homogenizer. After centrifugation for 10 Min at 3.000 rpm at 4°C (Eppendorf F45-30-10 rotor), supernatants containing equal amounts of total proteins were carefully adjusted to 45% sucrose (1.6 ml final volume) and pipetted at the bottom of an ultracentrifuge tube. Samples were then overlayed with 1.6 ml 30% and 0.8 ml 5% sucrose (in 150 mM NaCl; 20 mM Tris/HCl pH 7.4; 0.1 mM EDTA) to create a sucrose gradient. Samples were centrifuged for 16 h at 41.000 rpm at 4°C in a swing-out rotor (SW60Ti, Beckman Instruments), and seven fractions were collected starting from top and analysed by SDS-PAGE.

### Immunofluorescence

Hela cells grown on a coverslip were transfected using GeneJuice (Novagen). After 24 hours, cells were fixed in 4% paraformaldehyd in PBS for 15 minutes and blocked with 5% normal donkey serum for 1 hour. Permeablization was achieved by adding 0.2% Triton-X to the blocking solution. Cells were then incubated with primary antibody directed against V5-tag for 45 minutes at room temperature, washed extensively with PBS, and incubated with fluorophore-coupled secondary antibody for 45 min. Cells were mounted using ProlongGold (Invitrogen) and imaged with a Zeiss LSM710 confocal microscope equipped with a 63x/1.4 oil immersion objective.

## Results and discussion

Recently, it was suggested that the kidney disease protein podocin may exist in two isoforms that differ in size [6,7]. Whereas the larger canonical isoform (Pod^canon^) has been studied in detail, almost nothing is known about a shorter version of the protein (Pod^short^). To characterize this putative new isoform, we used database searches and found that the isoform corresponding to the short human isoform of podocin is also predicted in several other species all belonging to the order of primates. However, these primate non-canonical isoforms were predicted based on the presence of the human suggested isoform, and no cDNA or EST clones supporting the existence of these isoforms could be found in the databases. Since intron/exon boundaries are conserved between mouse and human, we speculated that a similar murine short isoform existed, yet no such EST could be found in our database searches. Moreover, several strategies to clone the short splice variant from mouse kidney and glomerulus cDNA libraries were not successful [data not shown]. Thus, we focused on the confirmation and identification of the short podocin isoform in human samples based on the predicted sequence of this isoform.

**Figure 2:**
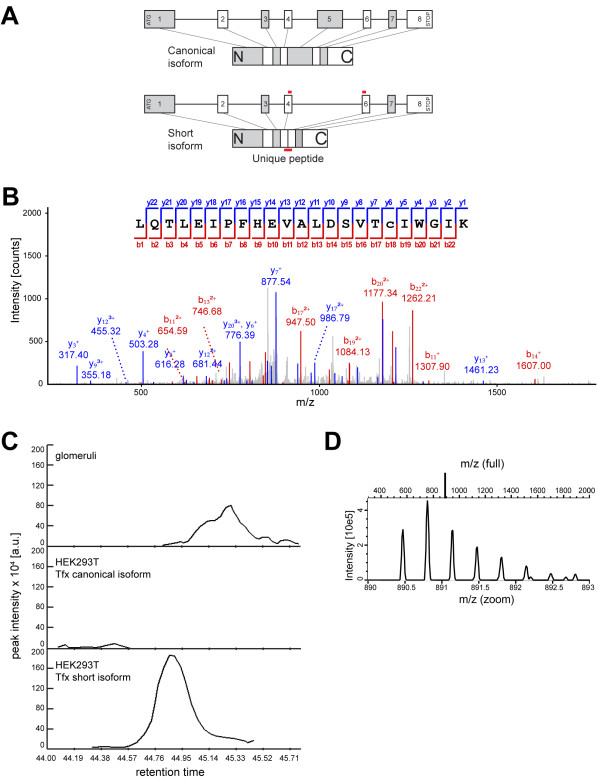
**Mass spectrometry proves the presence of the podocin short isoform in human kidney. A**) Schematic representation of the intron/exon structure of podocin and the resulting protein. The shorter isoform lacks exon 5. To prove the existence of the shorter isoform, we searched for a unique peptide expressed from the joined ends of exon 4 and 6. Tryptic peptides extracted from a SDS-PAGE gel area corresponding to the shorter isoform of podocin were analysed by nanoLC-ESI-MS/MS. **B**) MS2 spectrum of the isoform specific peptide with the sequence LQTLEIPFHEVALDSVTcIWGIK (m/z = 890.47). (The non-capitalized letter “c” denotes carbamidomethylation). The peptide was unambiguously identified in protein digest of HEK 293 T cells expressing the short isoform. **C**) Extracted ion chromatogram of the MS1 precursor masses of the isoform specific peptide from human glomeruli, and HEK293T cells transfected with the canonical or the short podocin isoform at the time of peptide identification. The chromatogram reveals the presence of the same mass in human glomeruli at a very similar elution time (m/z = 890.47). **D**) MS1 isotope pattern of the respective mass of the HEK293T sample transfected with the short isoform. The isotope pattern is consistent with a triply charged peptide.

To analyze the existence of the human short isoform further, we intended to demonstrate the presence of the shorter isoform on protein level using a mass spectrometric approach. To identify Pod^short^, we aimed to detect a peptide spanning the border between exon 4 and exon 6 as such a peptide would not be present in the canonical variant (Figure 2A). One crucial limitation of mass spectrometry based identification in human glomerular samples is the low purity of a preparation using the classical sieving technique as well as the limited amount of starting material. We therefore used lysates of HEK293T cells transfected with the canonical and short podocin isoform, respectively, as reference samples. These samples were processed in parallel to the glomerular sample and analyzed by mass spectrometry subsequent to the glomerular lysates as described in the method section.

Within the sample containing the short isoform of podocin, we unambiguously identified the isoform-specific peptide with the sequence LQTLEIPFHEVALDSVTcIWGIK. This triple-charged peptide carried a carbamidomethylation at the cysteine residue (m/z = 890.47). The MS2 spectrum for this peptide is depicted in Figure 2B.

Next, we analyzed whether the specific mass corresponding to this isoform-specific peptide (+/−10 ppm) was also found within a very limited time window (+/− 1 min) in the MS1 precursor chromatogram of the glomerular sample or the sample obtained from HEK cells expressing the canonical podocin isoform. As expected, the peptide mass was absent in the sample obtained from HEK293T cells transfected with the canonical isoform whereas many other peptides matching to podocin were by far more abundant in this sample. However, the mass corresponding to the short isoform specific peptide was also found in the human glomerular sample (Figure 2C). In addition, the MS1 precursor isotope pattern confirmed the presence of a triple charged peptide mass within both samples (Figure 2D). Combining these results, we report evidence on protein level for the existence of a shorter isoform of human podocin.

**Figure 3:**
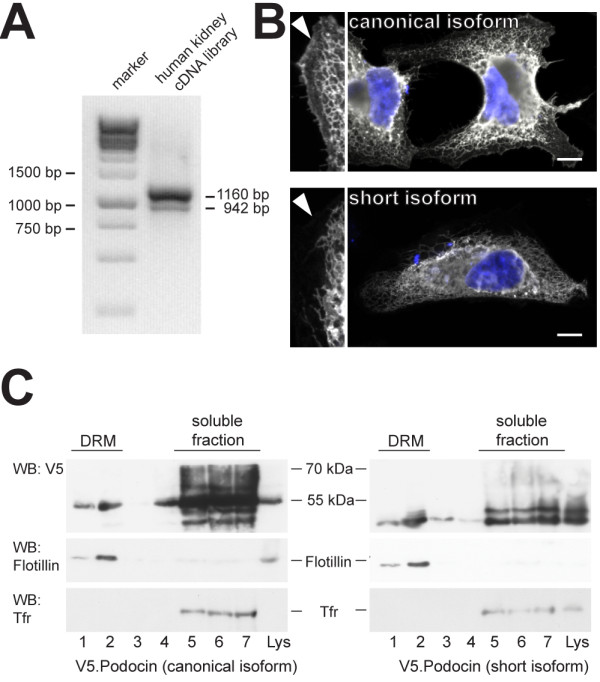
**The podocin short isoform is retained in the ER and is found in the detergent resistant membrane fraction. A**) PCR with podocin specific primers yields products for both isoforms from a human kidney cDNA library. **B**) Immunofluorescence staining of the canonical and the short isoform in Hela cells transfected with the corresponding constructs show that only the canonical isoform reaches the plasma membrane (arrowhead in the enlarged part of the image). The short isoform is retained in the endoplasmic reticulum. Scale bar = 10 μm. **C**) DRM association of both podocin isoforms. HEK293T cells expressing the respective V5-tagged podocin isoform were lysed in 1% TX-100 on ice and subjected to sucrose density gradient centrifugation. Fractions 1–7 were collected from the top and analyzed by Western blot. Both isoforms fractionate into the DRMs (fractions 1 and 2, as identified by flotillin staining). Antibodies against the transferrin receptor (TfR) and Flotillin-2 were used as markers for the Triton soluble and insoluble fractions, respectively.

**Figure 4:**
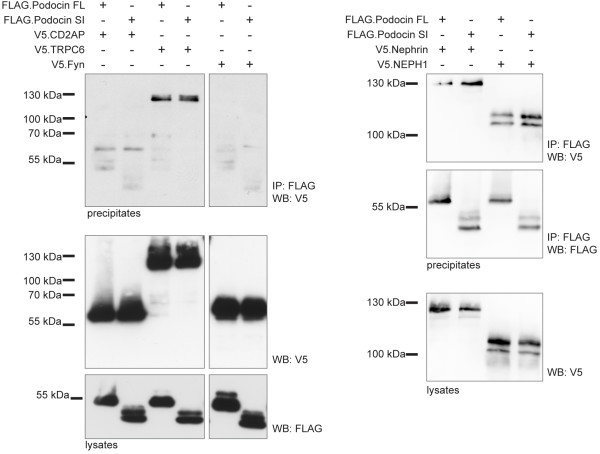
**The podocin short isoform interacts with known podocyte proteins.** CD2AP, TRPC6, neprin and NEPH1 co-precipitate with both podocin isoforms (FL, full length (canoncical isoform); SI, short isoform). FLAG- and V5-tagged proteins were expressed in HEK293T cells and precipitated with anti-FLAG antibody as indicated. Western blot analysis was performed with a V5 specific antibody. Expression levels of FLAG.podocin constructs in the lysates are shown below.

To elucidate protein function, we cloned Pod^short^ from a human kidney cDNA library (Figure 3A). The canonical isoform of podocin exerts its function at the slit diaphragm protein/lipid complex at the plasma membrane. We therefore tested protein localization of Pod^short^. Overexpression in HeLa cells and subsequent immunofluorescence staining revealed that the short isoform was retained in the endoplasmic reticulum and did not traffic to the plasma membrane (Figure 3B). Interestingly, many mutants of the canocial isform are also retained in the endoplasmic reticulum [15]. A second level of targeting occurs with the recruitment of podocin into detergent resistant membranes, which is defective in a variety of mutants [14]. We used sucrose density gradient ultracentrifugation to test if the short isoform fractionated into the detergent resistant membrane fraction (DRM). Contrary to the differences seen in subcellular localization, both podocin isoforms similarly localized to DRMs (Figure 3C), thus indicating that the cholesterol recruiting property was not compromised. Due to its interaction with several other slit diaphragm proteins and its localization to detergent resistant membranes, podocin is a central organizer of the slit diaphragm protein complex [5,16]. We examined the interaction of the Pod^short^ with several known slit diaphragm interactors in co-immunoprecipitation experiments. Despite the fact that the short isoform has a different subcellular distribution, we could not observe any difference between the interaction of the canonical and the short podocin isoform with CD2AP, TRPC6, NEPH1 and nephrin (Figure 4).

**Figure 5:**
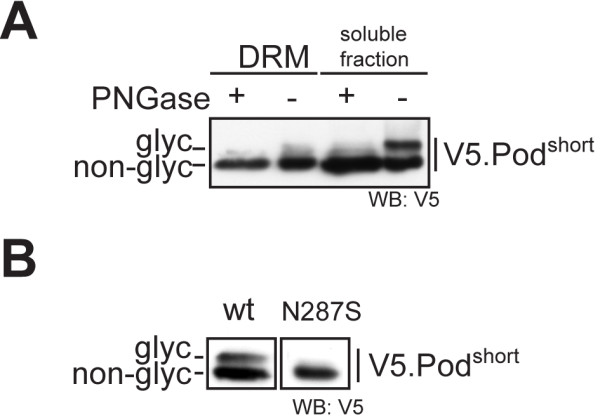
**The podocin short isoform is N-glycosylated. A**) PNGase-F treatment removes the double band from the short isoform in DRM fraction 7. Lysates from Figure 3 were subjected to treatment with PNGase-F and immunoblotted and detected with anti-V5 antibody. **B**) N to S mutation of the N-glycosylation consensus motif completely abrogates the formation of a double band. The asparagine at position 287 corresponds to amino acid 355 in the full-length protein. HEK293T cells were transfected with V5-tagged Podocin (short isoform or short isoform N287S, respectively) and lysates were immunoblotted and detected with anti-V5 antibody.

Lystes from cells transfected with Pod^short^, showed a prominent double band in Western blots (Figure 3C, fractions 5–7; Figure 4). We hypothesized that a part of the protein pool was post-translationally modified. One possible form of post-translational modification is N-glycosylation. For stomatin, another PHB-domain protein, point mutations have recently been identified that change the membrane topology to a transmembranous form with N-glycosylation occurring at the extracellular part [17]. The N-glycosylation of stomatin led to a well-detectable double band in western blots very similarly to the double band we observed with Pod^short^. We therefore tested next if the additional western blot band of the podocin short-isoform was the result of N-glycosylation. Treatment of lysates with PNGase-F, an enzyme that selectively removes N-glycosyl sugar chains, abrogated the second band in Western blot experiments (Figure 5A). To validate this finding further, we mutated the single N-glycosylation consensus motif (N-X-S/T, where X is any amino acid, except for proline) in the podocin short isoform (N287S [aa 355 in the canonical isoform]). Mutation of this motif from NRT to SRT completely prevented the occurrence of a second band in Western blots (Figure 5B), hence confirming our hypothesis that the observed double band was due to N-glycosylation of Pod^short^. It is interesting to note that deleting parts of the PHB domain as it occurs in Pod^short^ has a similar effect on the protein membrane topology as a single point mutation in a region preceding the PHB domain as in stomatin^P47S^. It has been shown that also in wildtype stomatin, the formation of the hairpin loop protein was not 100% efficient and that a certain pool of the wildtype protein was also N-glycosylated [17]. Because of the low abundance compared with the hairpin loop form, a physiological function for the transmembranous form was considered rather unlikely [17]. For Pod^short^, it seems that a considerable fraction is glycosylated and hence present in the non-hairpin loop conformation. However, the functional implication of these observations and the physiological role of Pod^short^ remains to be elucidated.

## Conclusions

Here we provide evidence for a human podocin splice variant that lacks exon 5. This splice variant is translated into protein but does not reach the plasma membrane in detectable amounts. The canonical isoform of human podocin has been studied extensively: It recruits a multimeric protein supercomplex – the slit diaphragm protein complex – to cholesterol-rich fractions of the plasma membrane thus providing the microenvironment needed for proper function of associated cation-channels and signaling at the slit-diaphragm. Since we did not find an EST or a cDNA corresponding to the short isoform from any other species, except from human, we wondered whether this isoform is transcribed and translated in humans and can be identified on protein level. Previous reports have indicated the presence of a protein corresponding to this isoform [6,7]. However these analyses were based on antibody techniques which can be difficult to interpret from complex samples such as glomerular lysates. We therefore used mass spectrometry to unambiguously confirm the presence of this short podocin isofrom in human glomerular lysates. This isoform still interacts with known interaction partners and, like the canonical isoform, partitions into detergent resistant membranes. However, it does not localize to the plasma membrane, but this naturally occurring variant is retained in the endoplasmic reticulum, a phenotype previously known from many podocin mutants. Interestingly, almost half of the short-isoform protein appeared to be N-glycosylated at an asparagine residue close to the C-terminus. This indicates that at least parts of the short isoform podocin shows a transmembranous conformation with the C-terminus facing towards the lumen of the endoplasmic reticulum. However, the functional implications of these findings remain elusive. It is an interesting observation that there is no database evidence based on ESTs or cDNAs for any other species indicative of the existence of this short isoform. Most likely the lack of such evidence in the primate species that have this isoform predicted is due to the lack of EST data. However, for other species as for example mouse or rat there is plenty of EST and cDNA data available. Yet this particular isoform cannot be found although intron/exon boundaries for exon 5 are conserverved. This may indicate that the described short isoform of podocin is specific to primates and missing in rodents.

## Abbreviations

DRM: Detergent resistant membranes; CID: Collision-induced dissociation

## Competing interests

The authors declare that they have no competing interests.

## Authors’ contributions

LAV was involved in all experimental steps and drafting of the manuscript. EMS and JT were involved in cloning steps. BAS was involved in the assessment of glycosylation. MMR, TL and DU designed the mass spectrometry strategy and carried out the analyses, also TL helped to draft the manuscript. BeS and EMS prepared the lysates for mass spectrometry. TB and MH designed and coordinated the study and drafted the manuscript. All authors read and approved the final manuscript.

## Pre-publication history

The pre-publication history for this paper can be accessed here:

http://www.biomedcentral.com/1471-2369/14/102/prepub
